# Epithelial Neutrophil-Activating Peptide (ENA-78), Acute Coronary Syndrome Prognosis, and Modulatory Effect of Statins

**DOI:** 10.1371/journal.pone.0003117

**Published:** 2008-09-03

**Authors:** Issam Zineh, Amber L. Beitelshees, Gregory J. Welder, Wei Hou, Nasser Chegini, Jun Wu, Sharon Cresci, Michael A. Province, John A. Spertus

**Affiliations:** 1 Center for Pharmacogenomics and Department of Pharmacy Practice, University of Florida College of Pharmacy, Gainesville, Florida, United States of America; 2 Department of Epidemiology and Health Policy Research, University of Florida College of Medicine, Gainesville, Florida, United States of America; 3 Department of Obstetrics and Gynecology, University of Florida College of Medicine, Gainesville, Florida, United States of America; 4 Division of Statistical Genomics, Center for Genome Sciences, Washington University School of Medicine, St. Louis, Missouri, United States of America; 5 Department of Medicine, Washington University School of Medicine, St. Louis, Missouri, United States of America; 6 Mid America Heart Institute, Kansas City, Missouri, United States of America; Leiden University Medical Center, Netherlands

## Abstract

Endothelial inflammation with chemokine involvement contributes to acute coronary syndromes (ACS). We tested the hypothesis that variation in the chemokine gene *CXCL5*, which encodes epithelial neutrophil-activating peptide (ENA-78), is associated with ACS prognosis. We also investigated whether statin use, a potent modulator of inflammation, modifies *CXCL5's* association with outcomes and characterized the *in vitro* effect of atorvastatin on endothelial ENA-78 production. Using a prospective cohort of ACS patients (n = 704) the association of the *CXCL5* −156 G>C polymorphism (rs352046) with 3-year all-cause mortality was estimated with hazard ratios (HR). Models were stratified by genotype and race. To characterize the influence of statins on this association, a statin*genotype interaction was tested. To validate ENA-78 as a statin target in inflammation typical of ACS, endothelial cells (HUVECs) were treated with IL-1β and atorvastatin with subsequent quantification of *CXCL5* expression and ENA-78 protein concentrations. C/C genotype was associated with a 2.7-fold increase in 3-year all-cause mortality compared to G/G+G/C (95%CI 1.19–5.87; p = 0.017). Statins significantly reduced mortality in G/G individuals only (58% relative risk reduction; p = 0.0009). In HUVECs, atorvastatin dose-dependently decreased IL-1β-stimulated ENA-78 concentrations (p<0.0001). Drug effects persisted over 48 hours (p<0.01). *CXCL5* genotype is associated with outcomes after ACS with potential statin modification of this effect. Atorvastatin lowered endothelial ENA-78 production during inflammation typical of ACS. These findings implicate *CXCL5*/ENA-78 in ACS and the statin response.

## Introduction

Endothelial inflammation plays a major role in the development and perpetuation of cardiovascular disease (CVD). The inflammatory process is complex, with various cellular mediators known to contribute [1], [2]. Multifunctional cytokines such as interleukin-1 (IL-1) play central roles in vascular inflammation. IL-1 produced from monocytes, macrophages, and other cells can initiate inflammation by inducing the production of chemokines from endothelial cells [3], [4]. Chemokines, in turn, propagate the process through recruitment and activation of additional cellular mediators of inflammation including neutrophils. This process is involved in many cardiovascular conditions including acute coronary syndrome (ACS). Moreover, previous work has established that monocyte chemokines such as monocyte chemoattractant protein-1 (MCP-1) are prognostic in ACS [5]. However, the role of neutrophil chemokines in ACS has not been well described.

*CXCL5* (MIM *600324 a.k.a. epithelial neutrophil-activating peptide 78 or ENA-78) is a C-X-C chemokine that attracts and activates neutrophils. Furthermore, *CXCL5* expression has been shown to be highly inducible in endothelial and vascular smooth muscle cells by IL-1β [6]–[9]. Recent data have implicated *CXCL5* and/or its receptors in congestive heart failure and ischemic stroke, making *CXCL5* a candidate gene for other manifestations of CVD including ACS [10]–[12]. We previously identified a −156G>C (rs352046) single nucleotide polymorphism (SNP) in the *CXCL5* promoter region to occur with high minor allele frequency in the general population and associate with both elevated plasma ENA-78 concentrations and leukocyte production of ENA-78 [13]. We therefore tested the primary hypothesis that this SNP is associated with 3-year all-cause mortality in a prospectively enrolled cohort of ACS patients.

Beyond testing this association, we also sought to describe potential associations with ACS therapies, namely statins, plausibly capable of influencing inflammation and outcome as a function of *CXCL5* genotype. Statins improve outcomes in ACS in part through anti-inflammatory properties. [14]. We previously reported that basal endothelial ENA-78 production is modulated by atorvastatin [15]. Therefore, we also tested the hypothesis that statin treatment may modify the association between *CXCL5* genotypes and outcomes in our ACS cohort. Finally, to offer insight into our epidemiological findings and validate *CXCL5* as a potential candidate in statin pharmacogenetics, we tested whether atorvastatin treatment modulates *CXCL5* expression and ENA-78 production from endothelial cells exposed to IL-1β, a model of cardiovascular inflammation typical of ACS.

## Results

### Clinical Characteristics

The mean patient age was 61±12 years, and the cohort was comprised of 36% women and 79% Caucasians. Complete clinical characteristics are displayed in Table 1. The minor allele frequency for −156C was 17%. Genotype frequencies did not deviate from Hardy-Weinberg expectations. The patients were similar when compared by *CXCL5* −156G>C genotype with the following exceptions: compared with G/G homozygotes, those with the C/C genotype were slightly younger, less likely to be Caucasian, had a greater prevalence of unstable angina as their ACS type, had higher admission DBP, had higher discharge HDL, and were less likely to be discharged on a statin (Table 1). G/C heterozygotes generally exhibited the above phenotypes in a fashion intermediate between G/G and C/C individuals. When baseline characteristics were compared by genotype in Caucasians alone, patients with the C/C genotype were older, but otherwise had no significantly different characteristics from the other genotype groups (Table 2).

**Table 1:** Baseline Characteristics.

	Overall	G/G	G/C	C/C	P value
	(n = 704)	(n = 498)	(n = 175)	(n = 31)	
Age	60.6±12.4	61.4±12.2	58.3±12.5	60.0±14.1	0.019
White	555 (79%)	437 (88%)	107 (61%)	11 (35%)	<0.0001
Women	253 (36%)	179 (36%)	60 (34%)	14 (45%)	0.51
ACS Type					
Unstable angina	284 (40%)	188 (38%)	75 (43%)	21 (68%)	
ST elevation MI	202 (29%)	154 (31%)	45 (26%)	3 (10%)	
Non ST elevation MI	215 (31%)	154 (31%)	54 (31%)	7 (23%)	0.042
Old LBB/Unknown	3 (0.4%)	2 (0.4%)	1 (0.6%)	0 (0%)	
History/risk factors:					
Dyslipidemia	403 (57)	297 (60%)	91 (52%)	15 (48%)	0.113
Diabetes	197 (28)	136 (27%)	50 (29%)	11 (35%)	0.604
Heart failure	55 (8)	32 (6%)	19 (11%)	4 (13%)	0.0955
MI	238 (34)	168 (34%)	58 (33%)	12 (39%)	0.832
HTN	459 (65)	323 (65%)	114 (65%)	22 (71%)	0.787
BMI (kg/m2)	29.6±6.3	29.4±6.0	30.3±7.1	29.4±7.2	0.215
Smoking					
Current	248 (35%)	168 (34%)	69 (40%)	11 (36%)	0.622
Former	258 (37%)	190 (38%)	56 (32%)	12 (39%)	
Never	197 (28%)	140 (28%)	49 (28%)	8 (26%)	
Disease severity					
EF (n = 670)	47.2±12.8	46.8±12.6	48.5±13.4	47.0±13.6	0.345
Admission SBP	136.6±27.0	136.4±26.8	135.9±26.7	144.4±31.0	0.257
Admission DBP	74.3±16.7	72.8±16.0	77.1±17.5	81.2±20.2	0.0009
Discharge total cholesterol*	178.7±42.7	176.4±40.6	184.7±47.8	181.0±43.0	0.118
Discharge HDL†	41.9±15.4	40.8±12.7	44.3±20.0	47.7±22.1	0.0097
Discharge LDL‡	102.9±37.3	100.7±34.6	108.3±43.0	107.2±41.8	0.0957
Discharge triglycerides§	176.8±122.7	181.3±131.7	169.3±99.4	141.3±71.5	0.228
Treatment strategy					
Medical management	259 (37%)	167 (34%)	75 (43%)	17 (55%)	0.048
PCI	414 (59%)	309 (62%)	92 (53%)	13 (42%)	
CABG	31 (4%)	22 (4%)	8 (5%)	1 (3%)	
Discharge statin	532 (76%)	390 (78%)	125 (71%)	17 (55%)	0.0044

*n = 603; ^†^n = 594; ^‡^n = 565; ^§^n = 593; ACS = Acute coronary syndrome; MI = Myocardial infarction; LBB = Left bundle block; HTN = Hypertension; BMI = Body mass index; EF = Ejection fraction; SBP = Systolic blood pressure; DBP = Diastolic blood pressure; HDL = High density lipoprotein; LDL = Low density lipoprotein; PCI = Percutaneous coronary intervention; CABG = Coronary artery bypass graft.

**Table 2:** Baseline Characteristics in Caucasians.

	All Caucasians	G/G	G/C	C/C	P value
	(n = 555)	(n = 437)	(n = 107)	(n = 11)	
Age	62.0±12.5	62.1±12.1	60.0±13.1	72.1±15.5	0.014
Women	194 (35%)	155 (35%)	32 (30%)	7 (64%)	0.073
ACS Type					
Unstable angina	204 (37%)	159 (36%)	40 (37%)	5 (45%)	
ST elevation MI	171 (31%)	137 (31%)	32 (30%)	2 (18%)	
Non ST elevation MI	178 (32%)	139 (32%)	35 (33%)	4 (36%)	0.958
LBBB/Unknown	2 (0.4%)	2 (0.5%)	0 (0%)	0 (0%)	
History/risk factors:					
Dyslipidemia	324 (58%)	263 (60%)	57 (53%)	4 (36%)	0.134
Diabetes	134 (24%)	110 (25%)	21 (20%)	3 (27%)	0.472
Heart failure	30 (5%)	25 (6%)	4 (4%)	1 (9%)	0.619
MI	179 (32%)	142 (32%)	32 (30%)	5 (45%)	0.560
HTN	346 (62%)	280 (64%)	59 (55%)	7 (64%)	0.231
BMI (kg/m2)	29.4±5.9	29.4±5.8	29.8±6.1	28.8±5.8	0.762
Smoking					
Current	180 (32%)	142 (32%)	35 (33%)	3 (27%)	0.766
Former	215 (39%)	174 (40%)	36 (34%)	5 (45%)	
Never	159 (29%)	121 (28%)	35 (33%)	3 (27%)	
Disease severity:					
EF (n = 532)	47.5±12.4	47.1±12.3	49.7±12.0	44.1±15.1	0.103
Admission SBP	134.2±24.6	134.6±25.0	133.3±23.5	129.1±22.3	0.695
Admission DBP	72.3±14.9	71.9±15.0	74.1±14.6	71.0±14.9	0.403
Discharge total cholesterol*	177.2±40.9	175.8±40.2	183.9±43.2	165.8±48.0	0.174
Discharge HDL†	40.6±14.8	40.2±12.3	42.3±22.6	38.8±10.3	0.452
Discharge LDL‡	101.5±34.9	100.1±34.0	107.5±36.9	100.8±49.0	0.224
Discharge triglycerides§	185.0±131.2	186.7±136.9	181.7±110.2	135.4±48.5	0.574
Treatment strategy					
Medical management	157 (28%)	132 (30%)	23 (22%)	2 (18%)	0.312
PCI	371 (67%)	283 (65%)	80 (75%)	8 (73%)	
CABG	27 (5%)	22 (5%)	4 (4%)	1 (9%)	
Discharge statin	439 (79%)	349 (80%)	82 (77%)	8 (73%)	0.665

*n = 473; ^†^n = 464; ^‡^n = 436; ^§^n = 465; ACS = Acute coronary syndrome; MI = Myocardial infarction; LBB = Left bundle block; HTN = Hypertension; BMI = Body mass index; EF = Ejection fraction; SBP = Systolic blood pressure; DBP = Diastolic blood pressure; HDL = High density lipoprotein; LDL = Low density lipoprotein; PCI = Percutaneous coronary intervention; CABG = Coronary artery bypass graft.

### Main Effect of CXCL5 Genotype on ACS Outcomes

**Figure 1:**
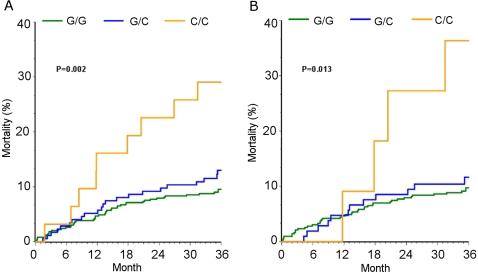
Kaplan Meier estimates for all-cause mortality by *CXCL5* −156 G>C genotype. Panel A represents the overall population; panel B represents the Caucasians only.

**Figure 2:**
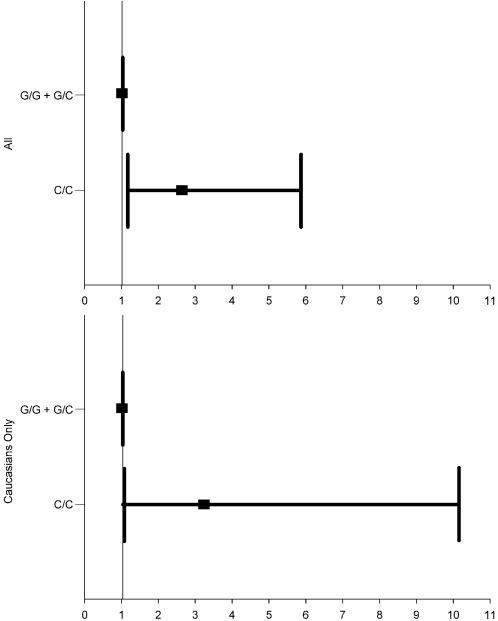
Adjusted hazard ratio and 95% confidence intervals for all-cause mortality by genotype. Top panel is overall population (p = 0.017) and bottom panel is Caucasians only (p = 0.043). Models adjusted for age, race, sex, ACS type, revascularization strategy, history of diabetes, and history of heart failure.

*CXCL5* −156 G>C genotype was significantly associated with 3-year all-cause mortality in ACS patients. The death rate was 10% in G/G, 13% in G/C, and 29% in C/C individuals (p = 0.005). C/C genotype was associated with a hazard ratio of 3.09 (95% confidence interval [CI], 1.54–6.19; p = 0.002) compared to G/C+G/G genotypes (Figure 1A). This effect remained significant when Caucasians alone were analyzed (HR 4.0, 95%CI 1.45–11.05; p = 0.008) (Figure 1B). The increased risk of mortality remained significant after adjustment for clinical covariates both in the overall population (HR 2.65, 95%CI 1.19–5.87; p = 0.017) (Figure 2A) and in Caucasians alone (HR 3.25, 95%CI 1.04–10.13; p = 0.043) (Figure 2B).

### Statin Interaction

**Figure 3:**
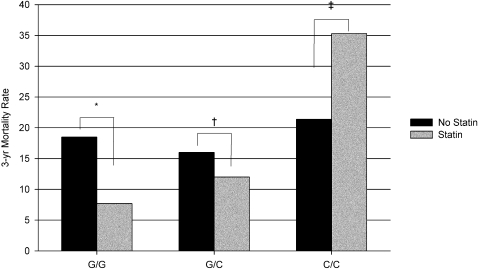
All-cause mortality rate by genotype among statin-treated and non-treated patients. *p = 0.0009., †p = 0.48; ‡p = 0.46.

We identified an interaction between discharge statin therapy and *CXCL5* −156 G>C genotype (p = 0.09). Individuals with the G/G genotype who were prescribed a statin at hospital discharge had a significantly lower 3-year all-cause mortality rate compared to those who did not receive a statin at hospital discharge (absolute risk reduction 10.8%; relative risk reduction [RRR] 58.4%; p = 0.0009) (Figure 3). Statin benefit was diminished in the G/C and C/C genotype groups. Patients with the G/C genotype experienced a non-significant RRR of 25% when discharged on statins (p = 0.48), while patients with the C/C genotype prescribed statin therapy had a numerically greater, although not significant, mortality rate compared to those not prescribed statin therapy (35.3% versus 21.4%, respectively; p = 0.46) (Figure 3).

### Effect of Atorvastatin on IL-1β-induced ENA-78 Production from Endothelial Cells

**Figure 4:**
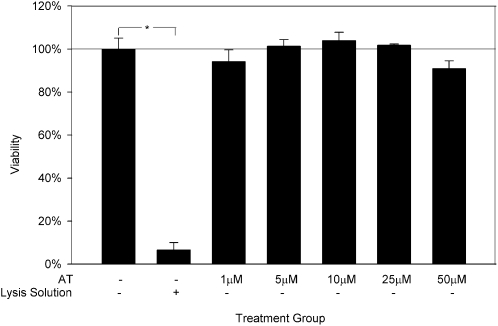
Cell viability of HUVECs cultured for 24 hours with atorvastatin. Data are presented as mean±SEM of 3 experiments. *p<0.0001. AT, atorvastatin.

**Figure 5:**
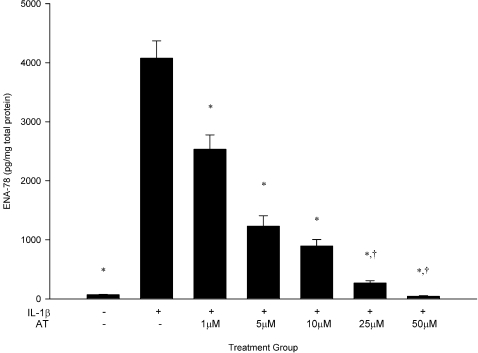
Atorvastatin attenuates IL-1β-induced ENA-78 production in a dose-dependent fashion. Data are presented as mean±SEM of 4 experiments. * p<0.0001 vs. IL-1β, †p = NS vs. control. AT, atorvastatin.

To validate *CXCL5*/ENA-78 as an endothelial target for statins, we utilized a HUVEC *in vitro* model of cardiovascular inflammation. There were no significant differences in HUVEC viability between control conditions and any dose of atorvastatin ranging from 1 µM to 50 µM (Figure 4). IL-1β increased ENA-78 production by nearly 60-fold relative to constitutive concentrations (4075±296 pg/mg vs. 69±7 pg/mg; p<0.0001; Figure 5). Treatment with atorvastatin attenuated this effect by 38%, 70%, 78%, 93%, and 99% in the five atorvastatin dose groups (p<0.001 for all comparisons vs. IL-1β).

**Figure 6:**
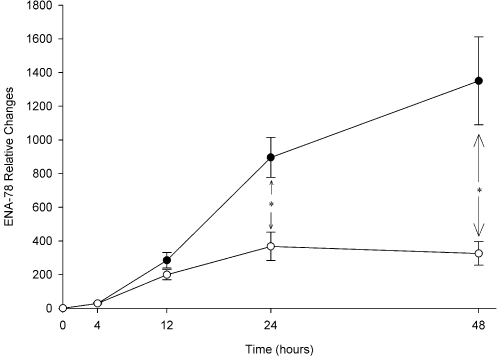
Atorvastatin attenuates ENA-78 production over time. Levels are relative to baseline (0 hour) for each condition. Data are presented as mean±SEM of 4 experiments. *p≤0.01. —○—, IL-1β stimulation+atorvastatin 10 µM; —•—, IL-1β stimulation alone; AT, atorvastatin.

The time-dependent course of ENA-78 changes relative to control in response to IL-1β and atorvastatin/IL-1β co-treatment are shown in Figure 6. Atorvastatin's time effect was significant at 24 hours and most pronounced at 48 hours. Specifically, atorvastatin attenuated IL-1β stimulated production of ENA-78 at 4, 12, 24, and 48 hours by 0%, 30%, 59% (p = 0.01), and 76% (p = 0.009), respectively.

**Figure 7:**
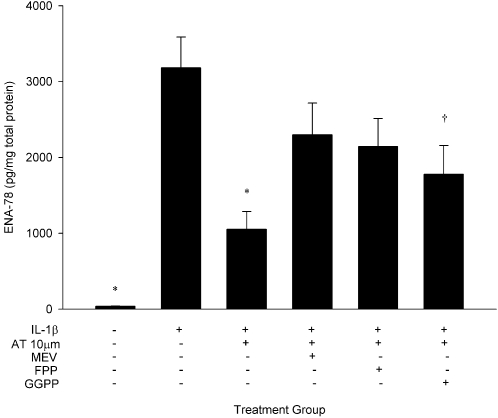
Atorvastatin effects on ENA-78 are reversed by mevalonate and its downstream metabolites. Data are presented as mean±SEM of 10 experiments. *p<0.001 and †p = 0.05 compared to IL-1β stimulation alone. AT, atorvastatin; FPP, farnesyl pyrophosphate; GGPP, geranylgeranyl pyrophosphate; MEV, mevalonate.

To determine whether atorvastatin effects were dependent on HMG-CoA reductase inhibition, HUVECs were cultured with IL-1β and atorvastatin 10 µM in the presence or absence of mevalonate. To assess whether downstream products of mevalonate biosynthesis are implicated in the drug effect, conditions also included cultures in the presence or absence of FPP and GGPP (Figure 7). Consistent with the previous experiments, atorvastatin significantly attenuated IL-1β-stimulated ENA-78 production (3181±407 pg/mg vs. 1054±234 pg/mg; p<0.001). Co-treatment with mevalonate and FPP reversed the effect of atorvastatin (p = NS vs. IL-1β), and GGPP partially reversed the effect of atorvastatin (p = 0.05 vs. IL-1β).

**Figure 8:**
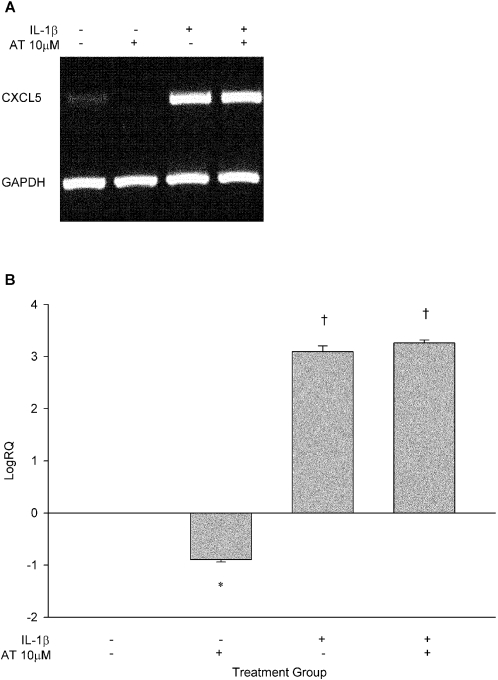
*CXCL5* expression is modulated by atorvastatin and IL-1β. (A) Gel electrophoresis of *CXCL5* and *GAPDH* PCR products; (B) Log_10_ relative quantification of *CXCL5* modulated by atorvastatin, IL-1β, and their combination normalized to *GAPDH* (N = 2 experiments). *P<0.005, †P<0.0001. AT, atorvastatin.

Atorvastatin significantly lowered constitutive expression of *CXCL5* (p<0.005), while IL-1β significantly induced *CXCL5* expression in HUVECs at 24 hours (p<0.0001). However, pre-treatment of HUVECs with atorvastatin did not blunt IL-1β induction of *CXCL5* expression (Figure 8).

## Discussion

In the current analysis, we demonstrate that a common promoter polymorphism in the ENA-78 gene (*CXCL5*) is associated with 3-year all-cause mortality in ACS patients, adding further support for a role of neutrophil chemoattractants in acute ischemic syndromes. Even after adjustment for age, sex, ACS type, revascularization strategy, heart failure, and diabetes, patients with two copies of the −156C variant allele were at 2.7-fold higher risk for mortality than patients with at least one copy of the −156G wild-type allele. The overall death rates for G/G, G/C, and C/C individuals were 10%, 13%, and 29%, respectively.

Importantly, we also demonstrated a pharmacogenetic relationship between *CXCL5* genotypes and discharge statin use. G/G patients experienced a significant RRR of 58% in all-cause mortality; G/C heterozygotes experienced a non-significant reduction of 25%; and C/C homozygotes experienced a non-significant increase of 39%. Our findings of an association between discharge statin use and mortality, as a function of *CXCL5* genotype, is an important extension of previous work. Several lines of evidence suggest that part of the cardioprotective effect of statins is related to their ability to modulate neutrophil function by various mechanisms, including reduced production of neutrophil chemokines 16–20. Our findings that the absolute mortality rate was numerically higher in C/C homozygotes discharged on a statin compared with C/C homozygotes not discharged on a statin is congruent with findings regarding the influence of statins on NAP-2, a neutrophil-activating chemokine exhibiting over 50% homology with ENA-78 [21]. Smith and colleagues demonstrated elevated NAP-2 plasma concentrations in patients with unstable angina [21]. However, while aspirin lowered plasma NAP-2, statin treatment stimulated NAP-2 production in patients, peripheral blood mononuclear cells, and platelets. This pro-inflammatory effect has also been observed by others; Kiener and colleagues demonstrated that some, but not all, statins stimulate monocyte production of pro-inflammatory cytokines including IL-8 (22% homology with ENA-78) *in vitro* and increase influx of leukocyte subtypes (including neutrophils) *in vivo* [22]. Therefore, while there is a strongly beneficial effect of statin therapy in the general population, some subsets of ACS patients may not derive such benefit. We have previously demonstrated *CXCL5* −156C carriers exhibit significantly greater plasma concentrations of ENA-78 and greater ENA-78 production from cultured leukocytes [13]. Therefore, it is conceivable that patients with the −156 C/C genotype may simultaneously represent a population at higher risk for ACS mortality that experiences little, if any, benefit from discharge statin therapy. The exact mechanism of this observation warrants further investigation.

In addition to our epidemiological analyses, we performed *in vitro* experiments and showed that *CXCL5*/ENA-78 is an endothelial target for statins. We corroborated previous work which showed ENA-78 is highly inducible by IL-1β in endothelial cells [6], [7], [9]. In the current experiments, atorvastatin modulated IL-1β-mediated ENA-78 production in a dose- and time-specific manner, through pathways involving HMG-CoA reductase and prenylation proteins. The drug effect began as early as 12 hours after stimulation and persisted out to 48 hours with no signs of diminishment. It has been hypothesized that statins exhibit early anti-inflammatory effects that may explain their benefit in acute manifestations of CVD (e.g., ACS). Furthermore, it has been shown that short-term statin treatment in certain cardiovascular settings result in demonstrable improvement in outcomes [23]–[25]. Our data suggest that early and persistent inhibition of IL-1β-induced endothelial ENA-78 production should be considered part of the spectrum of statin pleiotropism, and may represent an important mechanism of statin benefit in ACS and conditions marked by neutrophil involvement.

While postulated to be independent of LDL lowering, the anti-inflammatory effects of statins are thought to result from HMG-CoA reductase inhibition and/or inhibited production of prenylation precursors, such as FPP and GGPP. Our findings support this body of evidence in that atorvastatin's modulatory effect on ENA-78 was reversed by the addition of mevalonate and FPP, and partially reversed by GGPP. Previous studies have explored the effects of FPP and GGPP on statin responses, with the hope of elucidating the contribution of prenylation pathways on drug effects. Reversal of statin effects *in vitro* by the addition of FPP and GGPP implicate the Ras and Rho signaling pathways. Previous studies have shown that either Ras or Rho signaling may be involved in the statin effect, depending on the specific drug response tested [26]–[29].

While we showed atorvastatin to lower endothelial ENA-78 protein production under pro-inflammatory conditions, we also investigated whether atorvastatin lowered *CXCL5* expression. We found a significant reduction in basal *CXCL5* expression with atorvastatin treatment that was not observed for conditions with IL-1β stimulation. Nonetheless, we have previously shown basal endothelial ENA-78 protein production to be reduced by atorvastatin in a dose-dependent fashion, and currently extend this observation to a pro-inflammatory state [15]. There are several possible reasons for the disparate impact of atorvastatin on *CXCL5* expression in non-stimulated and stimulated conditions. Firstly, in a pro-inflammatory state (such as that induced by IL-1β), the predominant statin effect may be post-transcriptional in nature. Similar findings have been reported for the effect of rosuvastatin on MMP-7 production and expression whereby protein production but not gene expression was lowered [30]. A second explanation can be found from recent studies that implicate geranylgeranylation as an important homeostatic mechanism in IL-1β secretion from macrophages and peripheral blood mononuclear cells [31], [32]. Specifically, it was demonstrated that inhibition of isoprenoid production with statins led to upregulation of IL-1β. This effect was more pronounced upon addition of exogenous IL-1β (as in our experiments) suggesting that depletion of isoprenoids by statins shuts off the IL-1β negative feedback loop. As such, one can speculate that co-treatment of HUVECs with IL-1β and atorvastatin in our experiments may have potentiated IL-1β autocrine activity. Since *CXCL5* is highly inducible by IL-1β in HUVECs, the preponderant drug effect manifested as a reduction of ENA-78 protein concentrations (presumable through a post-transcriptional mechanism) with no impact on *CXCL5* expression.

There are several limitations to our analysis. Because of the epidemiological nature of the cohort study, data on white blood cell counts and differentials were unavailable. Therefore, the effect of these parameters on clinical endpoints in our population could not be directly tested. Along these lines, blood samples were not available to directly quantify ENA-78 protein concentrations in our patients. Nonetheless, we have previously shown that *CXCL5* genotype is a predictor of circulating ENA-78 concentrations and leukocyte production of ENA-78 [13]. Furthermore, the *CXCL5* genotype may in fact be a more stable marker of ACS risk than circulating ENA-78 concentrations since protein concentrations are more likely to be influenced by multiple stress-related factors. We have, in fact, shown this to be the case whereby endothelial nitric oxide (NO) synthase (*NOS3*) genotype was robustly associated with arterial stiffness, while circulating surrogate markers of NO activity (serum superoxide dismutase and nitrite) were not [33], [34]. We noted an interaction between discharge statin use and *CXCL5* genotype on outcomes. Because we did not have drug-specific information, we could not determine if this effect was differential influenced by the specific statin used, or the type of statin (i.e., hydrophilic vs. lipophilic); nor would we likely have the power to conduct such an analysis if the data were available. We also do not have information on the persistence of discharge statin use over time in these patients. Additionally, because of the small sample size for this subgroup analysis, there is a possibility of a false positive finding between genotype and discharge statin use. Nonetheless, others have estimated persistence of discharge statin use to be nearly 80% among patients similar in characteristics to our own [35]. In addition, our *in vitro* endothelial experiments validated ENA-78/*CXCL5* as an endothelial target of statin action in an inflammatory environment typical of ACS, adding biological plausibility to our epidemiological finding. Finally, because of the lack of granularity in the Social Security Administration Death Master File, the exact cause of mortality could not be determined. This is a known limitation of epidemiologic data and further analyses in independent cohorts are warranted. Despite these limitations, our data suggest that the ENA-78 gene, *CXCL5*, is associated with post-ACS all-cause mortality and is an endothelial target of atorvastatin *in vitro*. Whether ENA-78 modulation by statins is a central mediator of drug benefit in ACS is unknown. However, our findings strongly suggest a role of ENA-78 and *CXCL5* genetic variants as determinants of CVD and drug response.

## Methods

### Acute Coronary Syndrome Cohort and Gene/SNP Selection

The methods of accrual and adjudication for this prospective cohort have been previously described [36]. Briefly, patients with a confirmed ACS were enrolled from two Kansas City hospitals. Myocardial infarction (MI) was defined by elevated troponin level in combination with chest pain symptoms or electrocardiographic findings (ST-segment elevation or non-ST-segement elevation) consistent with MI. Unstable angina was defined by a negative troponin level and any one of the following: new-onset angina (<2 months), prolonged angina (>20 minutes) at rest, recent worsening angina, or angina that occurred within 2 weeks of MI [37]. Demographic information was obtained from patient interviews during hospital admission. Medical history, medication history, laboratory values, and inpatient treatment during the ACS hospitalization were obtained from chart abstractions. The Social Security Administration Death Master File (http://www.ntis.gov/products/ssa-dmf.asp) was queried approximately once yearly to obtain three-year all-cause mortality assessment. The study was approved by the institutional review boards of both institutions and written informed consent was obtained from all participants. Participants were genotyped according to previously published methods [38]. *CXCL5* (MIM 600324) was chosen as our *a priori* candidate gene based on 1) the known role of the neutrophil pathway in cardiovascular diseases including ACS [39]–[41], and 2) our previously published data demonstrating atorvastatin to significantly lower basal production of endothelial ENA-78 [15]. The −156G>C SNP (rs352046) was chosen as our candidate SNP because of our previous work demonstrating this SNP to be associated with variable ENA-78 protein concentration *in vivo* [13].

### In Vitro Endothelial Experiments

#### Cell Culture and Reagents

Human umbilical vein endothelial cells (HUVECs) (Lonza Walkersville, Inc., Walkersville, MD) were cultured as previously described [15]. Briefly, HUVECs between passes three and four were seeded at a density of 2.5×10^4^ cells per cm^2^ and cultured to 80% confluence with endothelial cell growth–supplemented medium. Once confluent, media were changed to serum free endothelial basal media for 24 hours prior to treatment after which basal media supplemented to 2% FBS concentration was used.

Treatments consisted of atorvastatin calcium (LKT Laboratories Inc., St. Paul, MN) 1–50 µM dissolved in dimethyl sulfoxide (DMSO). IL-1β, mevalonate, farnesyl pyrophosphate (FPP), and geranylgeranyl pyrophosphate (GGPP; all from Sigma-Aldrich, St. Louis, MO) were all dissolved in molecular grade water and used at concentrations of 2 ng/ml, 250 µM, 5 µM, and 5 µM respectively. All final DMSO concentrations were less then 0.1%.

#### Treatments

Cell viability was assessed by trypan blue staining. In experiments investigating the impact of atorvastatin dose on ENA-78 production, HUVECs were pre-treated with atorvastatin followed by addition of IL-1β two hours later. After dose-ranging studies were performed, the time-dependent effects of atorvastatin were investigated at 0, 4, 12, 24, and 48 hours. In separate experiments, cells were cultured with IL-1β and atorvastatin in the presence or absence of mevalonate, FPP, and GGPP to determine whether atorvastatin inhibition of ENA-78 production was dependent on HMG-CoA reductase inhibition and subsequent downstream pathways. Cell culture supernates for all experiments were stored at −20°C until analysis, which was performed within seven days.

#### Protein Quantification and Gene Expression

ENA-78 concentrations were measured and normalized to total protein content using cytometric immunofluorescence as previously described [15]. Ribonucleic acid (RNA) was isolated using a commercial kit (RNeasy mini kit, Qiagen Inc., Valencia, CA). Complementary deoxyribonucleic acid (cDNA) conversion was performed by high capacity cDNA reverse transcription (Applied Biosystems, Foster City, CA) per protocol using roughly 2 µg of total RNA. RNA quality was assessed by absorbance (Nanodrop, Nanodrop Technologies, Wilmington, DE). Real-time reverse-transcription PCR (RT-PCR) was performed using primer and probe sets for *CXCL5* normalized to the housekeeping gene glyceraldehyde-3-phosphate dehydrogenase (*GAPDH*; Taqman Gene Expression Assays, Applied Biosystems Inc.). Relative quantification was verified by gel electrophoresis of PCR products using exonic primers for *CXCL5* (5-CGGGAAGGAAATTTGTCTTGA-3; 5-AGCTTAAGCGGCAAACATAGG-3) and *GAPDH* (5-AAAATCAAGTGGGGCGATGCT-3; 5-GCCAGGGGTGCTAAGCAGTT-3). PCR for both *CXCL5* and *GAPDH* were performed in parallel reactions using the following conditions: 95°C for 2 minutes; 35 cycles of 95°C for 30 seconds, 59°C for 30 seconds, and 72°C for 30 seconds; followed by 7 minutes of elongation at 72°C.

### Statistical Analyses

#### ACS Cohort

Baseline characteristics were compared by ANOVA, student's t-test or chi-square tests as appropriate. Allele frequencies were calculated by allele counting and deviations from Hardy-Weinberg equilibrium were tested by chi-square. Death rates were compared by chi-square or Fisher's exact tests. To minimize multiple testing and maximize statistical power, one mode of inheritance was tested (recessive) only after visual inspection of survival curves for all three genotype groups. We had 80% power to detect a hazard ratio of 1.57 under the tested recessive model of inheritance, given our sample size of 704 participants and a minor allele frequency of 17%.

Cox proportional hazards models were developed that included the following pre-specified covariates: age, sex, ACS type (ST elevation MI, non ST elevation MI, or unstable angina), coronary revascularization strategy (medical management, percutaneous coronary intervention, or coronary artery bypass graft), heart failure, diabetes, and *CXCL5* genotype. Additionally, interactions between genotype and discharge statin use were assessed in the models. All models were conducted in the overall population and separately by race to minimize confounding by population stratification. Survival plots were generated by the Kaplan-Meier technique. The statin*genotype interaction term was considered significant if p<0.1 (two-sided) since multiplicative interaction testing is generally not powered to detect significance at an α = 0.05 [42]. In all other analyses, p<0.05 was considered significant. SAS version 9.1 was used for all analyses.

#### In Vitro Experiments

ENA-78 protein concentrations and *CXCL5* gene expression levels were compared for all conditions by one-way ANOVA with Tukey correction for multiple comparisons. An independent t-test was used to compare treatment differences at each time point in the time-course studies. Relative *CXCL5* gene expression was determined by the 2^−ΔΔCt^ method [43]. A p-value<0.05 was considered significant. Statistical analyses were performed with SPSS software, version 11.5 (SPSS Inc., Chicago, USA). The authors had full access to the data and take responsibility for its integrity. All authors have read and agree to the manuscript as written.
